# Rare Presentation of the Vein of Servelle in a Case of Klippel-Trenaunay Syndrome

**DOI:** 10.7759/cureus.57488

**Published:** 2024-04-02

**Authors:** Raju K Shinde, Rajat Mahawar, Sangita D Jogdand, Chetna Rathi

**Affiliations:** 1 Department of General Surgery, Jawaharlal Nehru Medical College, Datta Meghe Institute of Higher Education And Research, Wardha, IND; 2 Department of Pharmacology, Jawaharlal Nehru Medical College, Datta Meghe Institute of Higher Education And Research, Wardha, IND

**Keywords:** klippel-trenaunay syndrome, vein of servelle, short saphenous vein, parkes weber syndrome, limb hypertrophy, vascular malformation

## Abstract

Klippel-Trenaunay syndrome (KTS) is a rare congenital vascular syndrome involving bone and soft tissue hypertrophy of the involved limb and vascular malformations of the lymphatic, capillary, and venous systems. It is often confused with Parkes-Weber syndrome (PWS). KTS is characterized by a triad of capillary malformation in the form of port wine stains, bone or limb hypertrophy, and varicose veins. The vein of Servelle, also known as the lateral marginal vein, is one of the two persisting embryonic veins of the leg, the persistent sciatic vein being the other. Truncal vascular malformation can be a complication of failure of obliteration of these veins. We present a case of a 24-year-old male of KTS who had varicose veins in his right lower limbs since five years of age and macrodactyly with a synchronous presentation of the vein of Servelle.

## Introduction

Klippel-Trenaunay syndrome (KTS) is a rare disorder that affects the blood vessels and bones. It is characterized by three main symptoms: bone and soft tissue hypertrophy, cutaneous capillary malformation (port-wine stain), and congenital lymphatic and venous malformations (slow flow). KTS is not very common, with only about 1:100,000 people being affected by the condition [1]. KTS is typically diagnosed based on the presence of two out of three specific symptoms, which include abnormal skin coloration and overgrowth, abnormal bone or tissue growth, and varicose veins. The most common presentation of KTS is the presence of varicose veins and abnormal bone or tissue growth such as macrodactyly or limb hypertrophy, and they usually affect only one limb (75% lower limb) and 70% have incompetent vein extending from ankle to infrainguinal region [2].

KTS can be often misdiagnosed with its contemporary Parkes-Weber syndrome (PWS) and hence a proper diagnosis is warranted. The latter has arteriovenous fistulae, which is not a component of KTS. Diagnosis and management of KTS entails the involvement and participation of a multi-disciplinary team [1,2]. The vein of Servelle, also called the lateral marginal vein, is one of two persisting embryonic veins of the leg, the persistent sciatic vein being the other. Truncal vascular malformation can be a complication of failure of obliteration of these veins.

We present a case of a 24-year-old male of KTS who had varicose veins in his right lower limbs since five years of age and macrodactyly with a synchronous presentation of the vein of Servelle. Through this study, we wish to attract the focus of fellow colleagues on this rare entity.

## Case presentation

**Figure 1:**
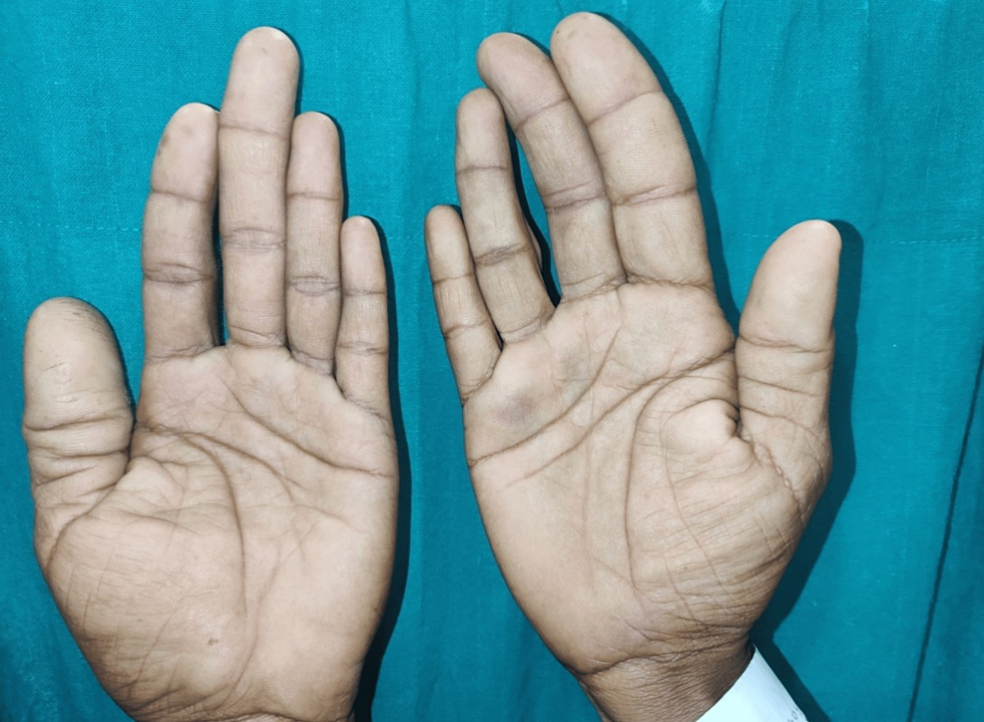
Macrodactyly

A 24-year-old male came with complaints of multiple swellings over the right lower limb since five years of age, which relieved on rest and aggravated on standing. He also complained of abnormally large fingers on both hands since childhood but more prominent since ten years and bleeding per rectum on defecation since 10 years. On presentation, the patient was conscious, cooperative, and well-oriented. On examination, there were no alterations in vital signs or state of consciousness. On examination of the upper limbs, the patient had unusually large fingers on both hands, predominantly the thumb, index, and middle fingers (Figure 1).

**Figure 2:**
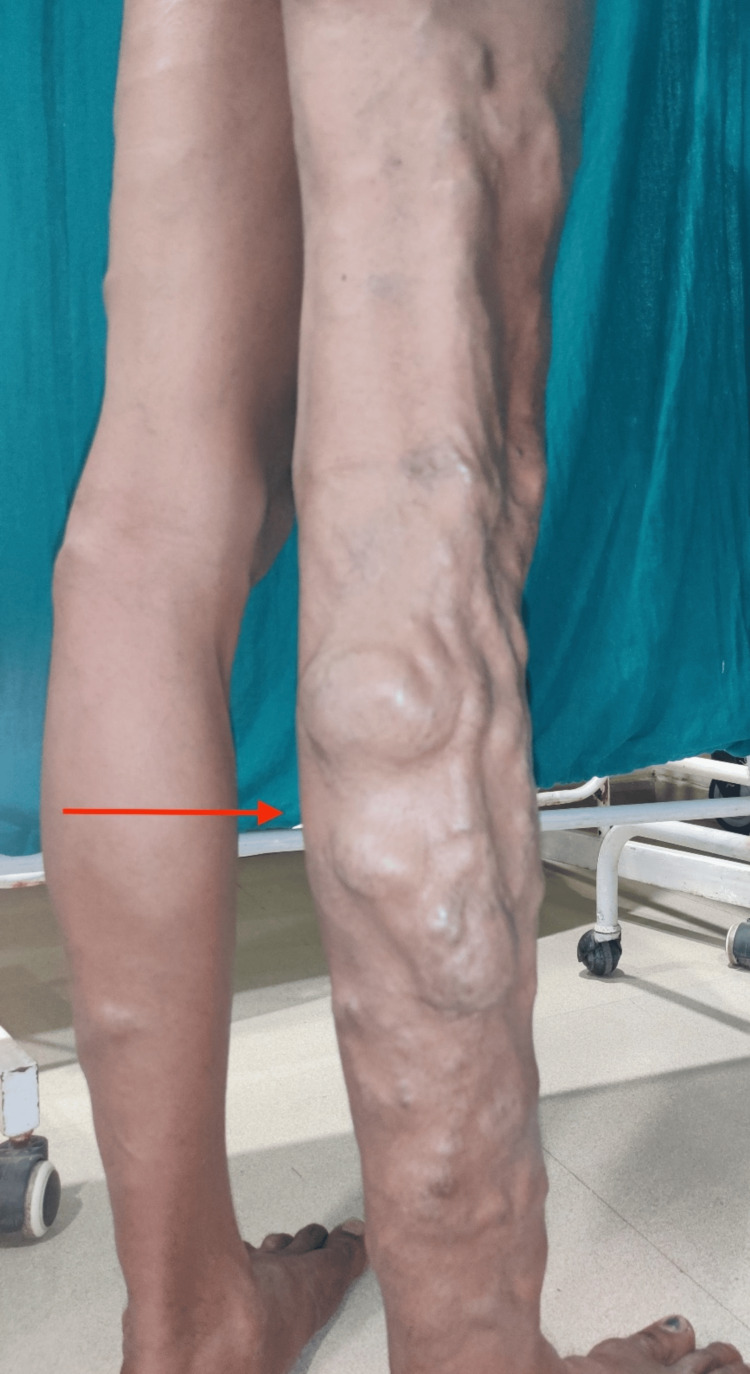
Varicose veins on the posterior aspect of the right leg

The examination of lower limbs revealed dilated, tortuous veins on the right lower limb below the level of the middle third of the right thigh. The dilated trunk was present on the posterior aspect of the leg till the lateral malleolus, indicating the involvement of the short saphenous vein (SSV) (Figure 2).

**Figure 3:**
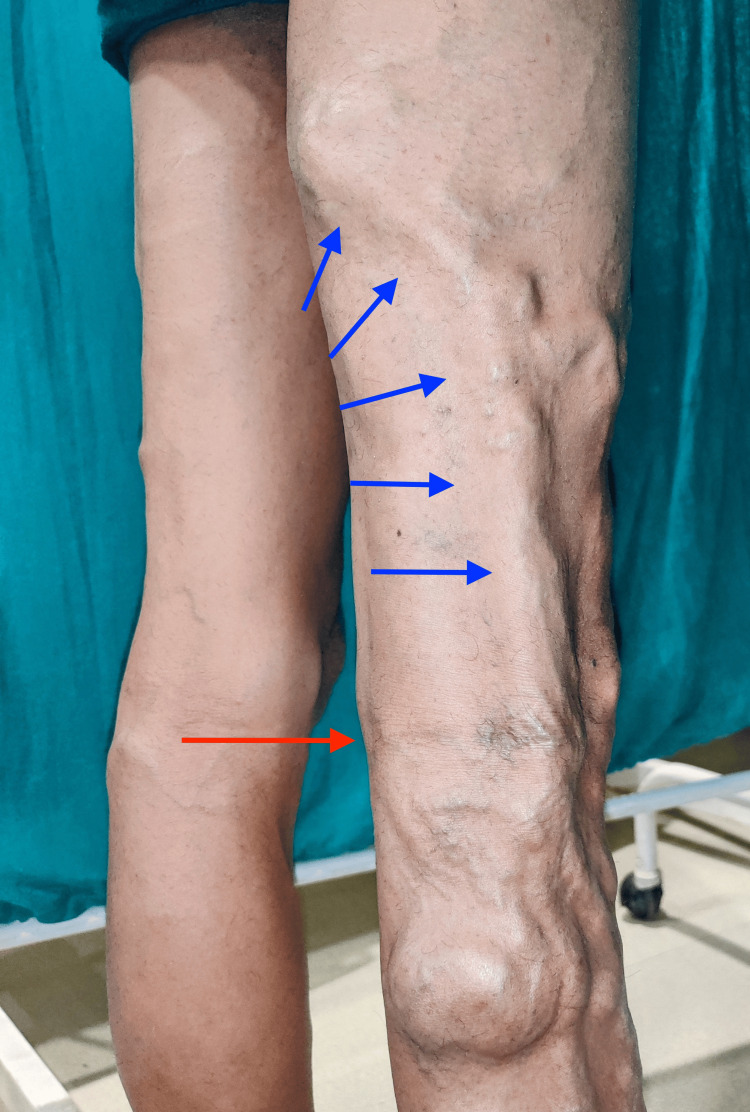
Lateral marginal vein of Servelle The red arrow depicts the knee joint and the blue arrows depict the lateral marginal vein.

The dilated trunk extended proximally to the posterior aspect of the middle thigh, indicating a possibility of an incompetent lateral marginal vein of Servelle (Figure 3).

**Figure 4:**
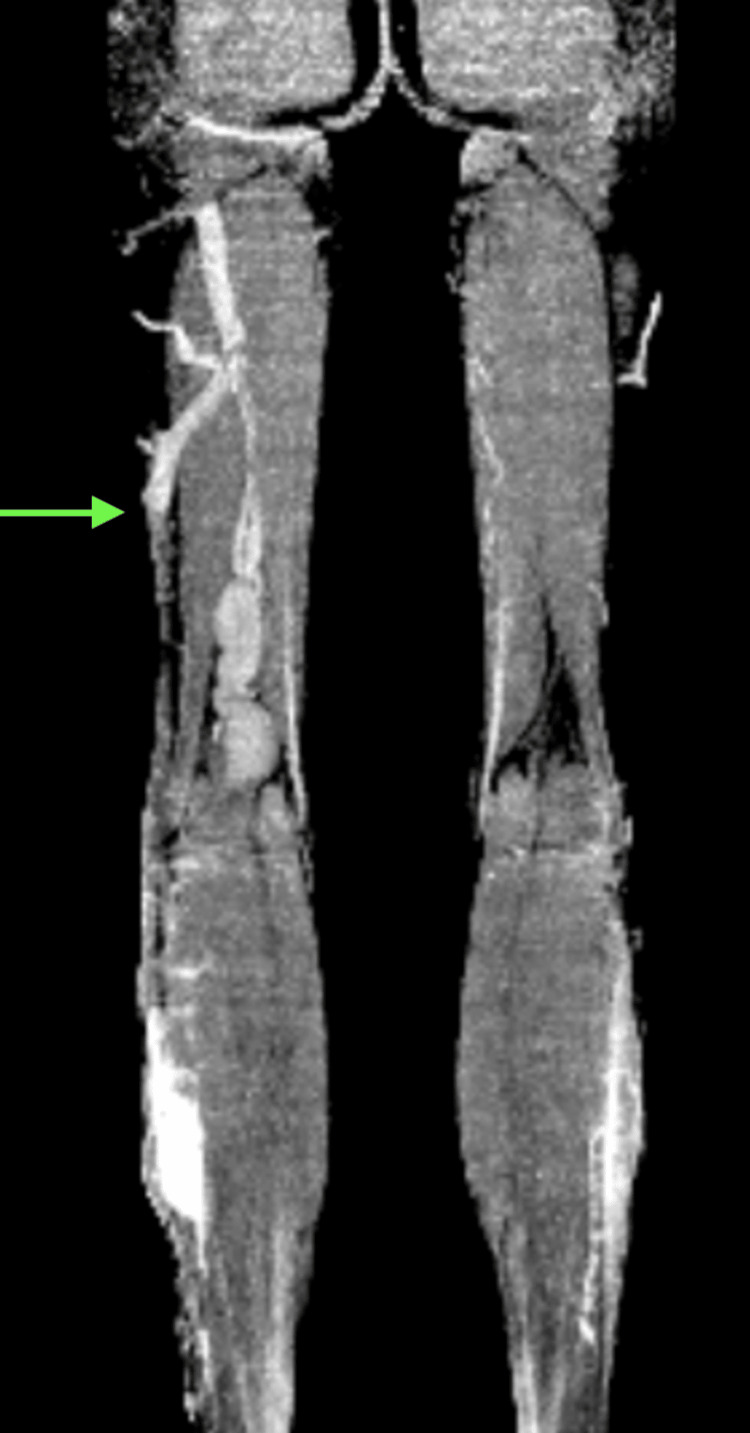
CT venogram of bilateral lower limbs showing the lateral marginal vein of Servelle (green arrow)

On digital rectal examination, no obvious abnormality was found. A Doppler study was done that prompted the diagnosis of an incompetent lateral marginal vein of Servelle. A CT venography scan confirmed the diagnosis of a lateral marginal vein of Servelle, as can be seen in Figure 4, and it also raised suspicion of rectal varicosities. The patient was given a clinical diagnosis of KTS with a lateral marginal vein of Servelle, treated conservatively for varicose veins without any complications, and kept on a three-month follow-up schedule.

## Discussion

KTS is a rare disorder that is characterized by abnormalities in the blood vessels, lymphatic vessels, and bones. It is caused by mutations in the PIK3CA gene and can cause overgrowth in some parts of the body. The severity of KTS can vary greatly from person to person, and it requires ongoing care from a team of medical professionals. KTS is typically diagnosed based on physical symptoms such as abnormal skin coloration and overgrowth, as well as abnormalities in the lymphatic and venous systems. Imaging tests may also be used to support the diagnosis [3]. French doctors Maurice Klippel and Paul Trénaunay originally identified KTS in the 1900s when they observed a connection between abnormalities in the blood vessels and abnormal growth of the limbs. The condition was originally referred to as "noevus variqueux osteohypertrophique." The syndrome is named after Klippel and Trenaunay [3,4].

KTS often affects the lower limbs but can also involve the upper limbs and trunk. The diagnosis of KTS is based on the presence of physical symptoms such as abnormal skin coloration and overgrowth, abnormalities in the lymphatic and venous systems, and overgrowth of certain parts of the body. Imaging tests or laboratory or genetic testing are not typically needed for the diagnosis of KTS [5]. In KTS, abnormal tissue growth causes enlargement of the limbs. The overgrowth of the extremities is usually noticeable at birth and does not typically progress significantly after that. KTS can affect the entire extremity, including the foot and hand, or it can cause isolated macrodactyly in an otherwise normal limb. Although KTS is sometimes confused with another condition called PWS, the two conditions are distinct. PWS is caused by a genetic mutation in the RASA1 gene and is characterized by high-flow vascular malformations, abnormal skin coloration, and overgrowth. Varicose veins occur in 72% of patients with KTS, the prominent feature being the persistent (embryonic) lateral vein present in 56% of patients, which can be considered a pathognomonic feature [4,5].

When KTS affects the lower extremities and pelvis, imaging tests may be used to evaluate the patency and size of the inferior vena cava (IVC) and iliac veins, as patients with KTS may be at risk for developing caval ectasia. Ultrasound is a useful tool for quickly identifying macrocystic lymphatic malformations (LMs) that may be infected or contain blood clots. CT may be used to detect chronic thromboembolic pulmonary hypertension or acute pulmonary thromboemboli. In patients with KTS, a leg length discrepancy (LLD) may occur, so regular monitoring of leg length may be necessary to prevent it. Non-operative management is an important modality in the therapy of symptomatic KTS. Usually, only patients who are refractory to drugs undergo surgical intervention. Patients with KTS are managed conservatively for varicosities with the use of graded compression garments, which can help reduce swelling, venous engorgement, and discomfort in the affected limbs. Drugs such as taselisib, which is a phosphatidylinositol-3-kinase (PI3K) inhibitor, have been used in the management of KTS but the safety profile of these drugs remains in question [6].

## Conclusions

KTS can be diagnosed clinically by an experienced physician since it is a disease of recognition, based on distinct clinical presentation and use of multiple modalities of imaging. The three main symptoms of KTS are varicosities and venous malformations, capillary malformations (port wine stains), and bone and soft tissue hypertrophy. To establish a phenotypical diagnosis of KTS, two of these three cardinal features are needed. There is no precise pathognomonic test to build the diagnosis of KTS, but the determination of the extensiveness and severity of the disease should be considered first. In our report, the patient presented with macrodactyly, varicosities, and had a persistent lateral marginal vein, which is not often recognized or documented. Treatment for symptomatic KTS patients often involves non-operative care. Generally, surgical intervention is reserved for individuals who are drug-resistant as surgical management can lead to worsening of symptoms. The lateral marginal vein does not warrant any change in the management course but should be identified and documented as a variant of the normal anatomy if present.

## References

[1] Jacob AG, Driscoll DJ, Shaughnessy WJ, Stanson AW, Clay RP, Gloviczki P (1998). Klippel-Trénaunay syndrome: spectrum and management. Mayo Clin Proc.

[2] Uihlein LC, Liang MG, Fishman SJ, Alomari AI, Mulliken JB (2013). Capillary-venous malformation in the lower limb. Pediatr Dermatol.

[3] John PR (2019). Klippel-Trenaunay syndrome. Tech Vasc Interv Radiol.

[4] Dervendizi Sikova D, Pavlova LT, V'lckova Laskoska MT, Nikolovska ST, Caca Biljanovska N (1999). Naevus varicosus osteohypertrophicus. An early diagnostic approach. Adv Exp Med Biol.

[5] Uller W, Fishman SJ, Alomari AI (2014). Overgrowth syndromes with complex vascular anomalies. Semin Pediatr Surg.

[6] Gloviczki P, Driscoll DJ (2007). Klippel-Trenaunay syndrome: current management. Phlebology.

